# Surgical Site Infection after Cesarean Delivery in Times of COVID-19

**DOI:** 10.1055/s-0041-1729144

**Published:** 2021-06-28

**Authors:** Vicente Sperb Antonello, Jessica Dallé, Ivan Carlos Ferreira Antonello, Daniela Benzano, Mauro Cunha Ramos

**Affiliations:** 1 Department of Prevention and Infection Control, Hospital Fêmina, Porto Alegre, RS, Brazil; 2 Medical School, Pontificia Universidade Católica do Rio Grande do Sul, Porto Alegre, RS, Brazil; 3 Universidade Federal do Rio Grande do Sul, Porto Alegre, RS, Brazil; 4 Sanitary Dermatology Outpatient Unity, State Health Secretariat, Porto Alegre, RS Brazil

**Keywords:** surgical wound infection, cesarean section, personal protective equipment, coronavirus, infecção da ferida cirúrgica, cesariana, equipamento de proteção pessoal, coronavírus

## Abstract

**Objective** To analyze effects of the COVID-19 pandemic on the consumption of personal protective equipment and products (PPEP), as well as the frequency of surgical site infection (SSI) among non-COVID-19 patients submitted to cesarean sections.

**Methods** A retrospective study was conducted in a maternity unity of a public teaching hospital which was not part of the reference service for COVID-19 treatment. It compared PPEP consumption and the occurrence of SSI after cesarean sections in monthly periods before and after the occurrence of the first case of COVID-19 in Porto Alegre, state of Rio Grande do Sul, Brazil. Personal protective equipment and products consumption was measured as units of masks, gloves, gowns, and caps, and use of alcohol-based products or soap for hand sanitation as ml/patient/day. The SSI index was calculated as the proportion of cases of SSI over the number of cesarean sections performed monthly during the study period.

**Results** There was an increase in all measured items of PPEP, with consumption of disposable masks with a median of 1,450 units in the pre-COVID period, and of 2550 in the post-COVID period (a 75.9% increase). A decrease of 49% in SSI was detected, with a median of 1.74 in the pre-COVID period and of 0.89 in the post-COVID period.

**Conclusion** The increase in consumption of PPEP could be a result of safer practices adopted by healthcare workers with the advent of COVID-19, of which the following reduction in the occurrence of SSI could be a direct consequence. Despite the severity of the crisis, one could state that extreme situations can lead to valuable reflections and opportunities for improvement.

## Resumo

**Objetivo** Analisar os efeitos da pandemia de COVID-19 sobre o consumo de equipamentos e produtos de proteção individual (EPPI), assim como a frequência de infecção de sítio cirúrgico (ISC) em pacientes não infectadas por COVID-19 submetidas a cesarianas.

**Métodos** Foi realizado um estudo retrospectivo em uma maternidade de um hospital público de ensino que não fazia parte do serviço de referência para o tratamento do COVID-19. Foram comparados o consumo de EPPI e a ocorrência de ISC após cesárea nos períodos mensais antes e após a ocorrência do primeiro caso de COVID-19 em Porto Alegre, RS, Brasil. O consumo de EPPI foi medido em unidades de máscaras, luvas, aventais e gorros, e o uso de produtos à base de álcool ou de sabonete para higienização das mãos em ml/paciente/dia. O índice SSI foi calculado como a proporção de casos de ISC sobre o número de cesarianas realizadas mensalmente durante o período do estudo.

**Resultados** Houve aumento em todos os itens medidos do EPPI, com o consumo de máscaras descartáveis apresentando uma mediana de 1.450 no período pré-COVID e de 2550 no período pós-COVID (aumento de 75,9%). Detectou-se também diminuição de ISC, com medianas de 1,74 no período pré-COVID e de 0,89 no período pós-COVID, com redução de 49% no valor da mediana.

**Conclusão** O aumento do consumo de EPPI pode ser resultado de práticas mais seguras adotadas pelos profissionais de saúde com o advento do COVID-19, do qual a redução na ocorrência de ISC pode ser uma consequência direta. Apesar da gravidade da crise, pode-se afirmar que situações extremas podem gerar reflexões valiosas e oportunidades de melhorias.

## Introduction

The pandemic of COVID-19 reached 30,675,675 cases and 954,417 deaths worldwide as of the 21 ^st^ of September 2020. Brazil was hit later but, as of the same date, 4,495,183 cases and 135,793 deaths were reported, with an underreporting of unknown magnitude. 1 The World Health Organization (WHO) and the Centers for Disease Control and Prevention (CDC) issued guidelines on personal protective equipment and products (PPEP) for healthcare workers during the care of suspected or confirmed COVID-19 infection. 2 3 Since the spectrum of the disease ranges from absence of symptoms to the demand of critical care, universal protection for healthcare workers in all settings became a standard of care. 4 5 6 Due to the high infectivity and potential morbidity and mortality associated with COVID-19, increasing adherence of healthcare workers to safety recommendations was foreseeable. 5 6 The present study aims at analyzing the possible effects of the COVID-19 pandemic on the consumption of PPEP, as well as the frequency of surgical site infection (SSI) among non-COVID-19 patients submitted to cesarean sections.

## Methods

A retrospective study was conducted in a public maternal hospital that was not part of the reference services for COVID-19 treatment, comparing PPEP consumption and the occurrence of SSI after cesarean sections in periods determined as pre-COVID and post-COVID, based on the occurrence of the first documented case of COVID-19 in our city. The PPEP consumption was measured as units of masks, gloves, gowns, and caps, and use of alcohol-based products and soap for hand sanitation as ml/patient/day. The SSI index was calculated as the proportion of cases of SSI over the number of cesarean sections performed monthly during the study periods. The SSI was based on the criteria established by the CDC, including cases of superficial incisional SSI, deep incisional SSI, and organ/space infections. 7 Data from April 2019 to February 2020 (pre-COVID) and from March to July 2020 (post-COVID) were compared. All cases of cesarean sections performed in both periods were included, except for five patients, in whom COVID-19 was suspected. Variables were described as median, minimum and maximum. Percentages were accompanied by confidence intervals (CIs). The analysis was performed with IBM SPSS Statistics for Windows, Version 20 (IBM Corp., Armonk, NY, USA). The present study was approved by the Hospital Ethical Review Board (registered under the number 50047715.9.0000.5530).

## Results

There was an increase in the consumption of all measured PPEP items (from 22.2% in the median value of consumption of surgical caps to 75.9% in the median of disposable masks), when comparing the pre-COVID to the Pos-COVID period ( Table 1 ). A decrease in the SSI was detected, with a reduction of 49% in the median of postcesarean SSI. The proportion of cesarean sections had no significant variation between both periods Pre-COVID and Post-COVID in the study.

**Table 1:** Consumption of personal protective equipment and products and surgical site infection after cesarean section index in the pre-COVID-19 and post-COVID-19 periods by healthcare workers

Factor	Pre-COVID *	Post-COVID **	% variation ***
Disposable masks (units/month)	1,450 (900–1,700)	2,550 (2,300–4,500)	75.9%
Disposable Gloves (units/month)	15,000 (13,000–17,700)	19,300 (17,000–22,000)	28.7%
Gowns (units/month)	140 (40–1,000)	200 (160–300)	42.9%
Caps (units/month)	1,800 (1,300–2,200)	2,200 (2,000–2,500)	22.2%
Alcohol-based products for hand hygiene (ml/pct-day/month)	22.90 (5.56–61.18)	33.96 (15.92–60.25)	48.3%
Soap for hand hygiene (ml/pct-day/month)	21.96 (6.11–42.25)	27.67 (17.66–30.65)	26.0%
Postcesarean SSI ((n of SSI/total of procedures) * 100)	1.74 (0–4.90)	0.89 (0.00–1.55)	48.9%
Cesarean	102 (92–126)	127 (105–150)	24.5%

Abbreviation: SSI, surgical site infection.

* Pre-Covid-19: April 2019 to February 2020.

** Post-Covid-19: and from March to July 2020. There was a significant increase in the absolute number of cesarean sections during this period because two maternity unitis in Porto Alegre were closed and obsteric cases were redirected to our hospital.

*** Date presented as median (minimum-maximum); %variation = ((final-initial)/initial)*100.

## Discussion

Being a retrospective study, a definitive cause-effect relationship cannot be established; nonetheless, the conspicuous association among the studied variables allows the inference that the decrease in consumption of PPEP was probably a result of the adoption of safer practices by healthcare workers after the advent of COVID-19, and that the reduction in the occurrence of SSI was probably its resulting consequence. Such a sizable reduction leads us to believe that the former adherence of healthcare workers was less than ideal, despite consistent education efforts. We believe that this reduction may resemble the one obtained by the introduction of hand washing proposed by Semmelweiss in the XIX century. 8

## Conclusion

Our data suggests that the advent of the COVID-19 epidemic had a positive impact on the adherence to safety measures and infection control among patients submitted to cesarean section. Despite the severity of the crisis, one could state that extreme situations can lead to valuable reflections and opportunities for improvement. A careful trend follow-up, after the eventual mitigation of the COVID-19, will be most needed.
